# Machine‐Learning Microstructure for Inverse Material Design

**DOI:** 10.1002/advs.202101207

**Published:** 2021-10-29

**Authors:** Zongrui Pei, Kyle A. Rozman, Ömer N. Doğan, Youhai Wen, Nan Gao, Elizabeth A. Holm, Jeffrey A. Hawk, David E. Alman, Michael C. Gao

**Affiliations:** ^1^ Materials Engineering and Manufacturing Directorate National Energy Technology Laboratory 1450 Queen Ave SW Albany OR 97321 USA; ^2^ ORISE 100 ORAU Way Oak Ridge TN 37830 USA; ^3^ LRST 1450 Queen Ave SW Albany OR 97321 USA; ^4^ Department of Materials Science and Engineering Carnegie Mellon University Pittsburgh PA 15213 USA

**Keywords:** machine learning, inverse problem, alloy design, microstructures

## Abstract

Metallurgy and material design have thousands of years’ history and have played a critical role in the civilization process of humankind. The traditional trial‐and‐error method has been unprecedentedly challenged in the modern era when the number of components and phases in novel alloys keeps increasing, with high‐entropy alloys as the representative. New opportunities emerge for alloy design in the artificial intelligence era. Here, a successful machine‐learning (ML) method has been developed to identify the microstructure images with eye‐challenging morphology for a number of martensitic and ferritic steels. Assisted by it, a new neural‐network method is proposed for the inverse design of alloys with 20 components, which can accelerate the design process based on microstructure. The method is also readily applied to other material systems given sufficient microstructure images. This work lays the foundation for inverse alloy design based on microstructure images with extremely similar features.

## Graphical abstract

This work reports the success of a very challenging task for machine learning images of extremely similar microstructure, demonstrating how artificial intelligence can potentially revolutionize an old science of metallurgy. More importantly, a useful inverse design method is proposed based on neural networks, which can design extremely complex alloys with 20 components and potentially better mechanical properties.

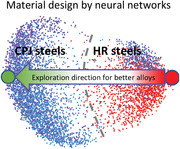

## 1 Introduction

**Figure 1:**
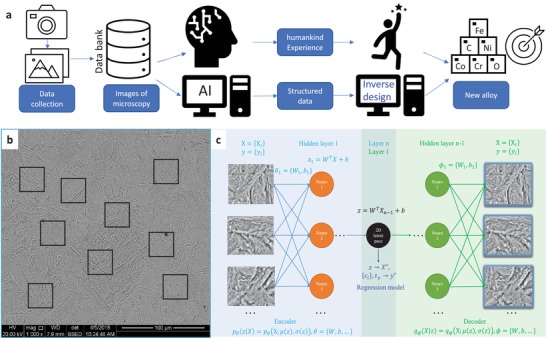
Machine learning for materials design. a) General procedure of material design with and without artificial intelligence (AI). The material design without AI strongly depends on humankind's experience, while AI offers new methods that are less prone to errors and biases. b) 32 sub‐images of 128 × 128 are randomly chopped from one representative scanning electron microscopy (SEM) image that has a martensitic microstructure. The bottom black bar is excluded from image chopping. (The black squares do not represent the real sizes of sub‐images). c) The schematic for the machine‐learning model (i.e., VAE) used in this study. The model consists of three sub‐models, i.e., the encoder model, the decoder model, and a regression model. The output of the regression model is basically the concentrations of the alloys, which is important to use in the next step for the inverse design scenario. In the training process, the images are both the inputs and labels with the aim of minimizing the difference between input and output images. After this process is completed, the core information is stored in the latent space (here 64‐dimensional), which is further reduced into two dimensions through kernel principal component analysis (PCA). The fine structure of hidden layers is not shown; the hidden layers are comprised of batch normalization layer, convolution layer, dropout layer, activation layer, etc.

Composition, processing, microstructure, and materials properties are the four cornerstones in materials research.^[^ ^1^ ^]^ Microstructures at various length scales can be obtained by various techniques, such as optical microscopy, scanning electron microscopy (SEM) and transmission electron microscopy, to name three, and these are the primary tools for characterizing materials and serving as the basis for explaining material behavior. Depending on the length scale, microstructure offers a rich font of information on the types of phases, their morphology, volume fraction and distribution, grain size, etc., as well as the disposition of various defects such as interfaces, twins, dislocations, stacking faults, etc. These defects and their thermodynamic and kinetic behavior help elucidate deformation processes and cooperative deformation mechanisms and further guide the design blueprint for new materials with targeted performance (**Figure** **1**a). Typical examples that show the importance of microstructure on mechanical properties include the widely acknowledged twin‐induced plasticity (TWIP) and transformation‐induced plasticity (TRIP) alloys.^[^ ^2^ ^]^ Optimizing the microstructure provides a pathway to overcome the well‐known strength‐ductility tradeoff.^[^ ^2^, ^3^ ^]^ Microstructure images are usually examined by individual researchers and the interpretation of microstructure‐mechanical behavior depends on the experience of those individuals. Recently, artificial intelligence (AI), or more specifically machine learning (ML),^[^ ^4^ ^]^ has been increasingly used in materials research to “read” the images and build a linkage between microstructure and the mechanical properties instead of relying on single‐point individual interpretations^[^ ^5^ ^]^ (Figure 1a). This creates new and exciting research opportunities using artificial neural networks (ANN). In addition to microstructures, ML methods have been successfully applied to many other types of data and topics in materials science,^[^ ^4c^ ^]^ such as training of structured data^[^ ^6^ ^]^ and corpus^[^ ^7^ ^]^ to learn thermoelectric,^[^ ^7a^ ^]^ thermodynamic^[^ ^6^, ^8^ ^]^ and mechanical^[^ ^6^, ^9^ ^]^ properties.

The aim of this study is to release the power of ML methods in the linkage of microstructure and properties that eventually results in a new method for inverse material design. Different from previous studies, we will address a particularly challenging problem in ML for similar microstructures, taking steels with a major phase of martensite as a prototypical example. The microstructure images for 9 to 12 wt% Cr ferritic‐martensitic steels, hereafter 9% Cr steel,^[^ ^10^ ^]^ are taken as input for a highly optimized ANN‐type algorithm based on variational autoencoder (VAE).^[^ ^11^ ^]^ Previously, VAE has been successfully used in several studies in materials science and physics.^[^ ^12^ ^]^ One of its outstanding advantages is that it allows for a flexible projection of the images into low‐dimensional space without the need of labeling the input data. This feature makes this algorithm particularly useful for a range of circumstances, including the design of new materials. Material design is core to materials science. It requires deducing the possible element mix needed to produce the requisite features that benefit material performance. In contrast to investigations of the properties of known materials, this is an inverse design process,^[^ ^13^ ^]^ which, by its very nature, is a very challenging task due to the asymmetry of information flow and the lack of constraints. Here, a neural network solution for the inverse material design has been proposed based on the knowledge obtained from microstructure images. The inverse design method can also be readily applied to other material systems given sufficient microstructure images.

## 2 Results

### 2.1 Microstructure Images in 2D Latent Space

ML methods are used to address the challenge of determining composition‐microstructure‐mechanical property using a prototypical example, i.e., the 9% Cr martensite/ferrite steels. The input data for the ML model consists of 621 SEM digital photomicrographs from 27 samples (designated HR and CPJ according to element concentrations) of 9% Cr steels of varying concentrations. The same alloy composition will result in the same microstructure when the processing parameters are controlled and kept the same. However, the actual process to synthesize an alloy can be affected by some environmental noises, which may introduce some uncertainties. A large training dataset of microstructure images can suppress the influence of unintentional factors since we have many of such images for the same alloy, which is beneficial to construct a successful data‐hungry model. The detailed processing procedures for the two series of alloys are described in the supplementary material as well as in.^[^ ^14^ ^]^ The images are first cleaned by removing the useless parts, such as the labels for information of the images generated by SEM; then each image is randomly subdivided into 32 sub‐images (Figure 1b). The ML model consists of three neural networks, each with a unique function (Figure 1c). The VAE algorithm (i.e., encoder and decoder neural networks) reduces the dimensionality of microstructure images (each≈1 million pixels) and extracts the specified features of interest from the images. Given the challenge posted by the similar microstructure, it is critically important to have the VAE model joined by a third regression model to help guide the distribution of the latent space. Careful tuning of neural‐network structures (number of neural layers and their types) and parameters (learning rate, etc.) is also critical to the success of the model. Additional details are contained in Supplementary Material.

**Figure 2:**
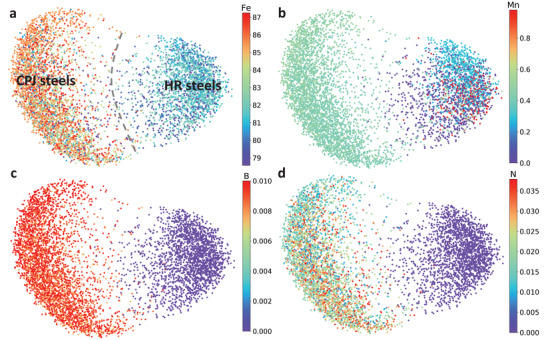
The twenty‐seven steels in 2D latent space. The samples reorganize into two distinct clusters. The left cluster is the CPJ steels (17 CPJ), while the right cluster is the HR steels (10 HR). The samples are labeled by the a) Fe, b) Mn, c) B, and d) N . Previous experimental experience is reflected by the Mo, B, and N labels that small variations of minor and trace elements matter. Here the much larger variation in Fe concentration cannot distinguish more clearly than these elements.

The dimensions of microstructure images are compressed by VAE and then reduced by kernel principal component analysis (PCA). This process is represented by the distribution of points in **Figure** **2**. The images populate latent space in a “heart” shape, which generally reorganize into two clusters according to the point density and the colors that represent the element concentrations. The right part of the “heart” belongs to the steels designated as HR, which were designed at an earlier stage and thus possess less than desired mechanical properties (i.e., yield stress and creep life, Figure S2, Supporting Information). The left part of the “heart” represents the CPJ designated steels with the improved mechanical properties, which were designed later than the HR ones. The clearly separated clusters strongly suggest the microstructure features in relation to the alloy composition are within the ability of the ML models and correlate with their mechanical properties.

The models also show how the element concentrations correlate with the steel samples. Although there are 20 elements in the 9% Cr steels, only some representative elements are shown in Figure 2, consisting of iron and three minor elements (Mn, B, N) of smaller atomic sizes. The minor elements, particularly the non‐metallic ones, usually have larger effects on microstructure and mechanical properties than their concentration in the steel might suggest. In Figure 2a, the HR steels have a lower Fe concentration (<84 wt% ), while the CPJ steels have a higher Fe concentration. The concentration of manganese in the HR series is either larger, or smaller, than ≈0.5 wt% in all the CPJ steels. This clearly shows how the time‐consuming trial‐and‐error approach identified the best concentrations for respective components. Figure 2b also shows the sub‐clusters within the right part of the heart for the HR steels, i.e., the upper sub‐cluster for medium Mn concentrations in cyan and the very high/low concentration in the lower part. Boron and N also influence the microstructure, which are captured by the ML models (see Figures 2c,d). Although the ML models capture the difference between the HR and CPJ steels, these differences in each cluster are very challenging to extract, particularly for the CPJ steels (Figures 2a,d, left part). The images are similar on average, so the points and the microstructures they represent occupy the same space with strong overlapping and with a certain degree of scattering. This requires the use of a larger dataset of images that is beyond the capability of models trained using 621 images.

The 9% Cr steels with varying constituents and associated mechanical properties were designed by a quasi‐Edisonian approach based on the best knowledge of 9% Cr steel behavior and CALPHAD assistance over 10 years with creep testing continuing. The ML model successfully groups the HR and CPJ steels into two distinct clusters and highlights the critical elements that strongly correlate with the sample locations in the latent space. These results lay the foundation for systematic model‐guided design of high‐performance steels.

### 2.2 Cr, Ni and Their Equivalent Concentrations

Our ML model also provides opportunities to check metallurgic concepts. Given the large number of elements in 9% Cr steel, the concept of concentration equivalents that combine elements having the same general effect on the microstructure may be relevant in labeling the microstructure images. Chromium and Ni are two key components for the 9% Cr steels. Here we consider both Ni and Cr concentrations and their equivalents to label the images. Several different methods to calculate the concentration equivalent have been published in the literature^[^ ^15^ ^]^ and the Ni/Cr equivalents defined by Hull^[^ ^16^ ^]^ are adopted.

**Figure 3:**
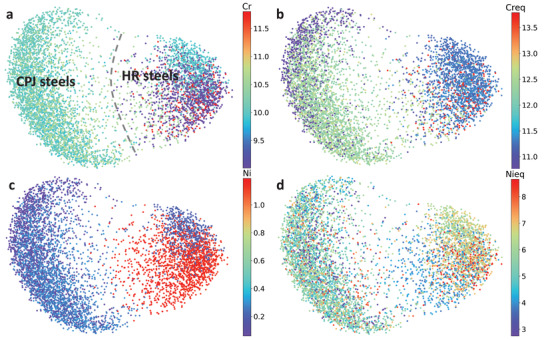
The Cr/Ni and their equivalent concentrations. The 27 samples labeled by the concentration using a) Cr, b) Cr equivalent (Creq), c) Ni, and d) Ni equivalent (Nieq).

**Figures** **3**a,b show Cr and Cr equivalent concentrations as the labels in the 2D latent space using the ML model. The equivalent concentration of Cr is 1–2 wt% higher than the concentration of Cr, which as a label can nicely separate the CPJ steels from HR steels. Also, sub‐clusters are visible in the HR steels, which is similar to what was seen for Mo. The top right corner for HR steels corresponds to the steel samples with a medium Cr concentration, while samples with either higher or lower concentrations are in the bottom right corner. Instead, the CPJ steels have approximately the same concentration of 10.5 wt% that may be further optimized in future design iterations. The Cr equivalent as a label can also separate the HR and CPJ steels, but it cannot show the sub‐clusters in the right portion of the data. Instead, it demonstrates some sub‐clusters in the left portion for CPJ steels. The Ni concentration as a label shows two clusters and two sub‐clusters (Figure 3c), such as the lower Ni region (in purple) and higher Ni in the lower portion for CPJ steels. In the right part for HR steels, the lower Ni region is in the upper portion, while the higher Ni concentration region is in the lower portion. The Ni equivalent changes several features of the distribution, including a shift of the maximum value. Unfortunately, it cannot clearly reflect the finer structure of the latent space but only some insignificant clusters, such as the right upper portion in yellow for 6.5 wt% Ni equivalent. Given these differences, Ni is a better label than Ni equivalent.

### 2.3 Order Parameter to Describe the Microstructure Features in 2D Space

**Figure 4:**
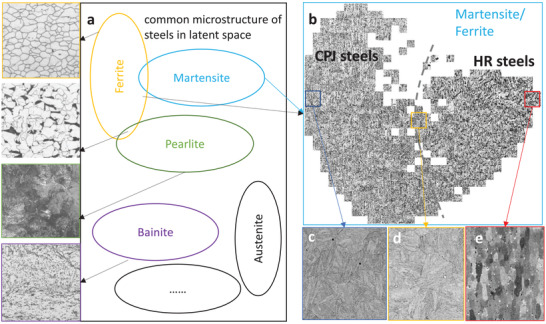
The common microstructure of steel in the latent space. a) A schematic showing the common microstructure in the ML latent space with representative ones to the left. The representative images are taken from reference.^[^ ^18^ ^]^ and references therein. There have been numerous studies addressing the differences between various phases (inter‐phase level). b) The different morphologies of martensite and ferrite are successfully differentiated in the 2D space, which is the latent space of VAE dimension‐reduced by the kernel PCA. The first component (horizonal direction) of the interpretable kernel PCA can be deemed as an order parameter to describe the feature of microstructure. This is at a much finer level and is more challenging due to the similarity of the same phase. c–e) Examples of the representative microstructure in the extreme left, middle and extreme right of the latent space.

To better show how the ML model reorganizes the microstructure images of the different steel samples, the data points in the latent space are replaced by the images that they represent. In **Figure** **4**a, the most common microstructure is shown in the latent space schematically. There have been numerous ML studies to differentiate the various phases in a microstructure, i.e., at the inter‐phase levels. Given the eye‐catching differences between various phases, this is not difficult to do with limited image data. However, few ML studies can differentiate similar microstructure morphologies. With the experimental data of twenty‐seven 9% steels accumulated in the past 15 years, the VAE model fine‐tuned in this research is able to successfully differentiate the microstructure among very similar compositions. This is shown in Figure 4b, with representative images in Figures 4c–e. Details on the microstructure morphologies are publicly available elsewhere and not restated here.^[^ ^17^ ^]^ One key detail worth mentioning is that a kernel principal component analysis (kPCA) is used to further reduce the VAE latent parameters instead of *t*‐distributed stochastic neighbor embedding (*t*SNE). The latter approach cannot well capture the gradual and continuous changes in microstructure, although it is always able to separate whatever images are fed into it, regardless of similarities.

In order to further check the predictability of the method, two representative steels (CPJ1 and HR68) were removed from the training data and a new model was trained. The new model presents an almost identical distribution of the microstructure images as seen in Figure 4b. The redefined model was then used to predict the concentrations of the two 9% Cr steel groups. The predicted concentrations and the experimental ones are in good agreement (see Figure S4, Supporting Information), particularly for the elements such as Fe, where the magnitudes in wt% are unambiguously predicted. These results render the present dataset a suitable example as a proof of concept in demonstrating the method described herein for inverse material design.

### 2.4 Inverse Material Design

**Figure 5:**
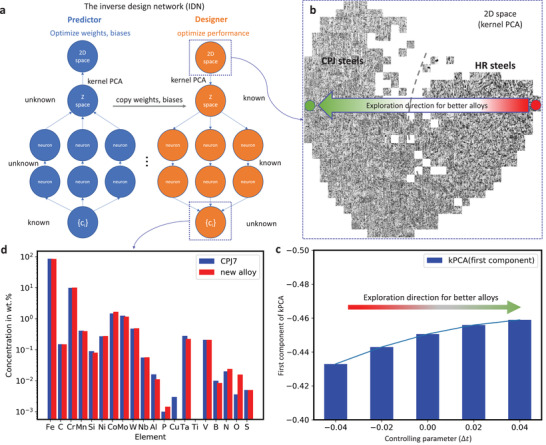
Inverse material design. a) Schematic for the inverse design network (IDN) for alloy design. It consists of a predictor network and a designer network. The two neural networks have different optimization aims. b) Exploration of the 2D latent space for the design of a better alloy, in this case a 9% Cr steel. The first component of kernel principal component analysis (kPCA) contains the most important information and can be interpretated as an order parameter of the microstructure. Indicated by the arrow, the first component of kPCA can be used to guide the design of new alloys. c, d) show the design process and the proposed concentration of elements for the 9% Cr steel as validation of proof of concept.

One of the ultimate aims of material research is to design new alloys with better performance. To do so, a neural network solution is proposed here to find a potentially optimal constitution through the inverse design procedure (**Figure** **5**). The 9% Cr steel is used as a proof of concept. Described in Figure 5 is an inverse design network (IDN). The procedure is to first train the regression model along with the VAE model. At this step, the weights and biases of the IDN are unknown. After feeding the element concentrations and the latent parameters of VAE, optimal weights and biases of IDN are obtained. Given a specific concentration of the 9% Cr steel, its distribution can be predicted in the latent space. As such, it is referred to as a predictor. In the next step, the weights and biases of the trained neural network are fixed but the input and output parameters are reversed. This allows the concentrations of elements to be calculated from the 64‐dimensional latent space, which actually realizes the design process in an inverse manner. The design process is guided by the 2D kernel PCA (kPCA) space, where the optimal direction is to decrease its first component (horizonal arrow in Figure 5b). It is worth mentioning that kPCA is not part of the regression model, but it is an important part of our inverse design scenario that will be elaborated below.

Mathematically, the optimal points in ***z***‐space can be found by the first derivative of a function *f* (***z***) =  kPCA(***z***), which is however very complex and not straightforward. The outperforming alloys and their *f*(***z***) correspond to smaller first components in 2D space. In this process, i) an optimal direction in the 2D space (Figure 5b) is defined from the rightmost position (i.e., red point for one HR steel, denoted by *f*(***z***_1_)) to the leftmost point (*f*(***z***_0_)). The aim at this juncture is to find the points in ***z***‐space which follow this direction to go beyond the left boundary of the known steels. Then, ii) starting from one optimal ***z***_0_, sample the ***z*** space around it using a prescribed step size and record the new ***z***. Subsequently, iii) the IDN is used to get the concentrations for the optimal ***z***, which is the essential information for the design of a new steel.

As an example, we consider a search routine defined as ***z *** = ***z***_0_  + Δ*t*(***z***_0_ − ***z***_1_) with Δ*t* as the controlling parameter in ***z*** space (i.e., the 64‐dimensional ***z*** space, not 2D *f*(***z***) space). The controlling parameter Δ*t*  =  0 corresponds to the green point in Figure 5b. For Δ*t*  =   − 0.04, −0.02,  0.02 and 0.04, the *f*(***z***) is plotted in Figure 5c. With the optimal direction in the 2D space as the guidance, two points/steels are identified that potentially outperform the known steels. The steel concentration at Δ*t*  =  0.04 is shown in Figure 5d. Compared to the starting CPJ4 steel, the newly proposed steel slightly fine tunes the concentrations of several elements (Figures 5d and S5, Supporting Information). Several element concentrations are reduced (i.e., Mn, Si, Co), while other elements are increased (i.e., Ni, Mo, W, etc.). The element concentrations of this new steel are closer to that of CPJ7 steel [29, 30] with variations as noted (Figure 5d). It is worth mentioning that the exploration in ***z***‐space is independent of the initial concentrations (here CPJ4). The validation of this prediction requires further experimental effort. Such guidance is extremely valuable given the fact that the sense of tuning component concentrations in complex alloys is not hypothetical and artificial but depends on a highly optimized neural network. The guidance is independent of researchers’ experience and the procedure can be unambiguously repeated.

### 2.5 Discussion

The similar microstructure images have been differentiated by a highly optimized neural network. The success of this very challenging machine‐learning task demonstrates how artificial intelligence can potentially revolutionize the old science of metallurgy. The key to this success lies in the design of the neural network structure and the optimal parameters. In principle, the VAE algorithm can optimize the distribution of images in the latent space when the training data volume is sufficiently large. However, given (i) the limited microstructure images and (ii) their extreme similarity, the VAE model alone cannot achieve this aim, which is aided by a third regression model here. The regression model takes a full consideration of all available information to guide the latent‐space distribution, connecting the components and latent space. It is also the regression model that triggers our new alloy design method. To optimize the performance of the neural networks, we find the regression model must have a higher weight than VAE (100:1) to decide how the points in the latent space to distribute. The optimal number is determined by tests.

The traditional trial‐and‐error method has been unprecedentedly challenged in the modern era when the number of components and phases in advanced alloys keeps increasing. The rise of high‐entropy alloys is one of the most representative examples of this trend.^[^ ^2^, ^3^, ^19^ ^]^ Usually, it is the researchers’ biased background and preferences that ultimately lead the alloy design process to a final chemistry. It is extremely challenging to find the optimal components in the huge and complex space of alloy candidates. The new neural‐network method for inverse material design proposed here can conquer this challenge, powered by artificial intelligence. After extracting the useful information from hundreds of images, the method can provide material candidates with optimal targeted properties, such as yield stresses.

## 3 Conclusions

In summary, a machine‐learning investigation relating microstructure images with chemistry and select mechanical properties was performed utilizing neural networks. The highly optimized model, which consists of a variational autoencoder and a regression model, reveals the relationships among these images in a straightforward manner. To the best knowledge of the authors, this is the first ML study to successfully differentiate similar microstructure, which at its core is different from previous studies that focused on very phases with significant and critical features. The challenge in differentiating performance with respect to microstructure lies in the extreme similarity of these steel images, where differences seem insignificant to the viewer. The success of the approach is a result of highly optimized neural network structures and fine‐tuned parameters therein. The model clearly identified the key role of several elements in the prototypical 9% Cr steel through the formation of the different features of the martensite steels. As such, a new inverse alloy design method is proposed based on neural networks. It demonstrates a systematic approach to design new alloys. The method developed and prescribed herein has the potential to replace the traditional trial‐and‐error approach currently used by offering high‐quality guidance that is independent of researcher bias. This work lays the foundation for a microstructure‐guided design of high‐performance alloys using information currently available.

## 4 Experimental Section

### Data Collection

A FEI Inspect F SEM was utilized to image and record digitally planar sections as well as creep fracture surfaces. The accelerating voltage was 10 or 20 keV. Backscatter electron (BSE) imaging modes were used in the inspection investigations. A minimum of 20 images were taken over an area of 0.1 mm × 0.12 mm with 0% overlap between images. Specimens were sent to a third party for planar surface polishing and etching.

### Data Processing

The data of SEM images consists of twenty‐seven samples, all of which are 9% Cr ferritic‐martensite steels of varying chemical concentrations. According to the concentration ranges, the steels were roughly classified as the HR and CPJ steels (just using these names to differentiate them). There are about 23 microstructure images available for each designated steel sample, totaling 621 images without overlapping microstructure information.

The images are cleaned by removing any non‐useful parts, such as the labels for information on the images generated by SEM. As the next step, an image is randomly subdivided into 32 sub‐images (Figure 1a). More specifically, a location is randomly located on the image and a sub‐image, 128 × 128 px^2^, is chopped. This results in 19872 images in total, 70% of which are used in training data for ML with the remaining 30% used for prediction. The SEM images are in grey scale, so the three color‐channels are occupied by identical numbers, and any of the three channels can be used as the feature. However, special attention must be given to the different color representations (or data types) used for the color channels. Some data types are 0–255 (int8) while others are 0–65535 (int16). The numbers are renormalized into 0–1 for convenience as well as to avoid potential biases.

The original SEM images have low contrast with biased brightness, as can be seen from their statistical data (Figure S1, Supporting Information). The images with low contrast and biased brightness result in useless correlation in the ML model, providing no contribution to the scientific causality. The serious consequences of these biases include the linear correlation in the ML model, which weakens the ability of ML models to learn from useful information. There are various methods to mitigate the problem. One way is to preprocess the images using methods like equalization before feeding them into ML models. The equalized image has better contrast with the maximum of the histogram for pixels located around 128, i.e., half of 256. Another method is to add one more layer of batch normalization in the neural network. Batch normalization can solve the bias problem as well as accelerate the learning rate. As such, this approach was selected.

### Machine Learning Models

The ML model consists of three neural networks, each of which has its own unique function (see Figure 1b). The VAE algorithm is used to reduce the dimensionality of microstructure images (each image is ≈1 million pixels) and extract the relevant features. The VAE model consists of two sub‐models, i.e., the encoder model and the decoder model. The encoder model transforms the input images *X* into unobserved latent variables *z* (here with size 64), represented by two vector parameters in the latent space (****μ****, ****σ****). More specifically, ***z *** =  ****μ**** + *ε*⊙ ****σ****, where ****σ**** is a random normal tensor. The dimensionality of the parameter ***z*** is further reduced if it is larger than two. This equation allows data (images) to be generated with similar features to the training images by mapping the latent space points back to the original input images. The latter is realized by the so‐called decoder model with a similar neural network structure as the encoder model. The fine structure of either encoder or decoder is comprised of multiple layers of various functions. Important functional layers include batch normalization, convolution, dense, pooling, activation, etc. The extracted low‐dimensional information is further compressed using a kernel principal component analysis (kPCA) method and then fed into a regression model. The regressed model takes elemental concentrations as its labels. Since the relationship between microstructure images and their features (e.g., alloy element concentrations, processing conditions, and properties) are complex, the regression model can make the best of the available information to guide the differentiation ability of the latent space.

The total error is comprised of the encoder‐decoder error *L*_VAE_ (KL divergence +cross entropy error) and the regression model *L*_reg_. Here, KL divergence refers to the Kullback‐Leibler divergence. After testing the total error function, *L*  =  *L*_VAE_ + 100*L*_reg_, gives satisfactory compromised accuracy of the regression model and quality of the latent space to represent the key information of the images. The Adam algorithm, with a learning rate of 1  ×  10^–3^, is used to optimize the error function for 100 epochs. A longer training time does not improve the accuracy of the models substantially. The implementation of the above algorithm is realized in the framework of TensorFlow with GPU acceleration.

## Conflict of Interest

The authors declare no conflict of interest.

## Author Contributions

Z.P. designed and performed the machine learning study, and wrote the manuscript. M.C.G. conceived and led the project. Z.P. designed the neural networks and performed modeling training and analysis. K.A.R. and O.N.D. designed the alloys and prepared the SEM images for machine‐learning models. Y.W., N.G., E.A.H. joined the analysis of the data and helped shape the direction of this study. J.A.H. and D.E.A. helped finalize the manuscript. All of the authors discussed the research, edited the manuscript, and approved its final version.

## Supporting information

Supporting Information

## Data Availability

Supporting Information is available from the Wiley Online Library or from the author.
