# Supplementary material for: Machine‐Learning Microstructure for Inverse Material Design
Source: Adv Sci (Weinh). 2021 Oct 29;8(23):2101207. doi: 10.1002/advs.202101207 (PMC8655171; doi:10.1002/advs.202101207)
Supplement: Supplementary file 1 — Supporting Information [file ADVS-8-2101207-s001.pdf]

© 2021 Wiley-VCH GmbH

ADVANCED SCIENCE
Open Access

Supporting Information

for *Adv. Sci.,* DOI: 10.1002/advs.202101207

Machine-learning microstructure for inverse material design

Zongrui Pei[1,2], Kyle A. Rozman[1,3], Ömer N. Doğan[1], Youhai Wen[1], Nan Gao[4], Elizabeth A. Holm[4], Jeffrey A. Hawk[1], David E. Alman[1], Michael C. Gao[1]

Supplementary Material to *Machine-learning microstructure for inverse material design*

Extended figures are provided to supplement the information in the main text

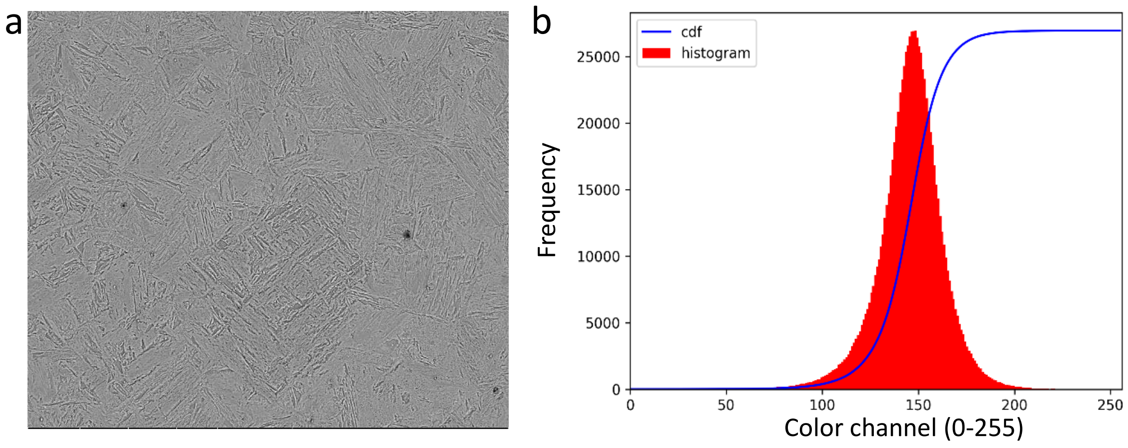

Figure S1. *The original SEM images have low contrast and biased brightness, as can be seen from their statistical data in the histograms.*

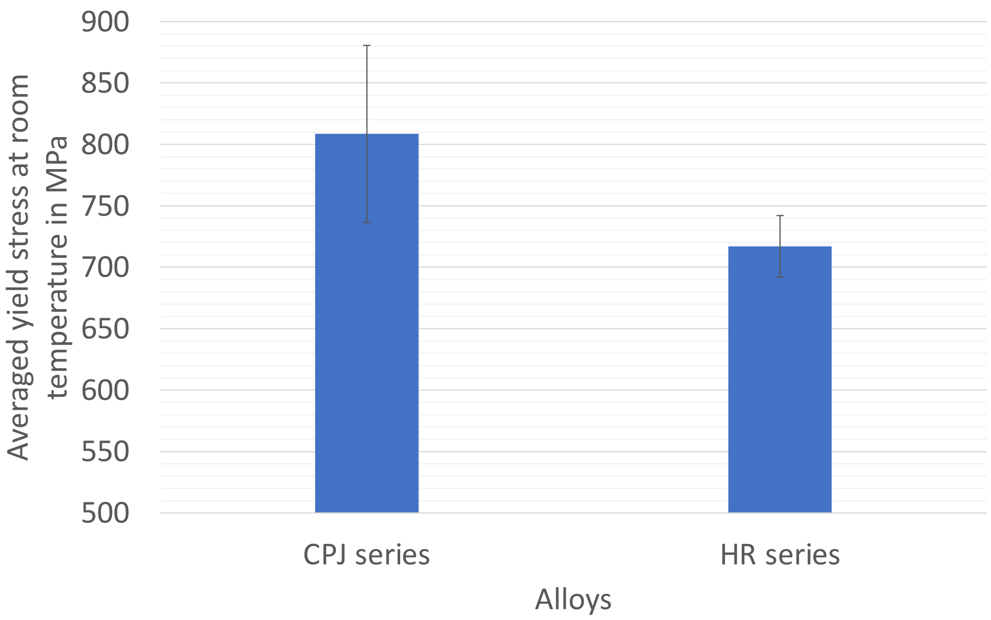

Figure S2 *The average yield stresses for CPJ series and HR series at room temperature. The value for CPJ is averaged over all 17 samples, while for HR series it is based on three samples of HR 52-54 for which room-temperature yield stresses are available.*

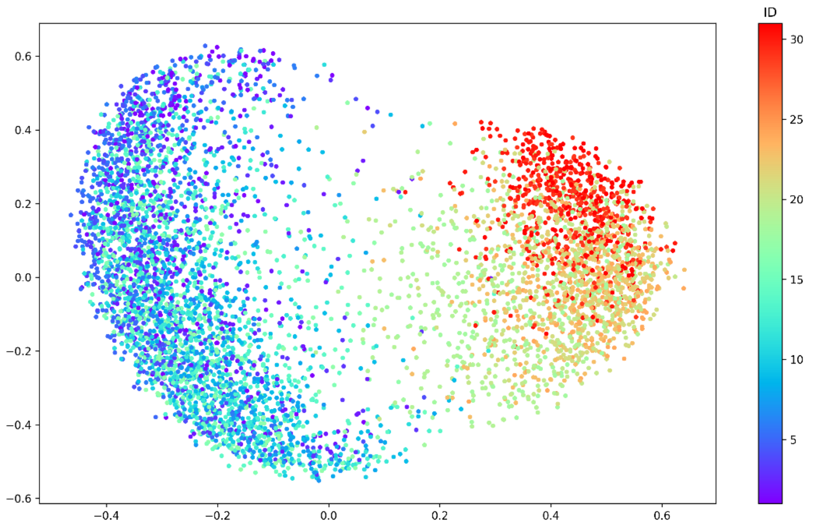

Figure S3. *The images are labeled by their sample ID in the reduced two-dimensional space. Each 9% Cr steel sample is randomly given a number as to their identity (ID. CPJ steels have IDs 1-17 while HR steels have IDs 18-27. The first 17 samples constitute the left cluster of the "heart" shape, with the remaining 10 samples constituting the right cluster.*

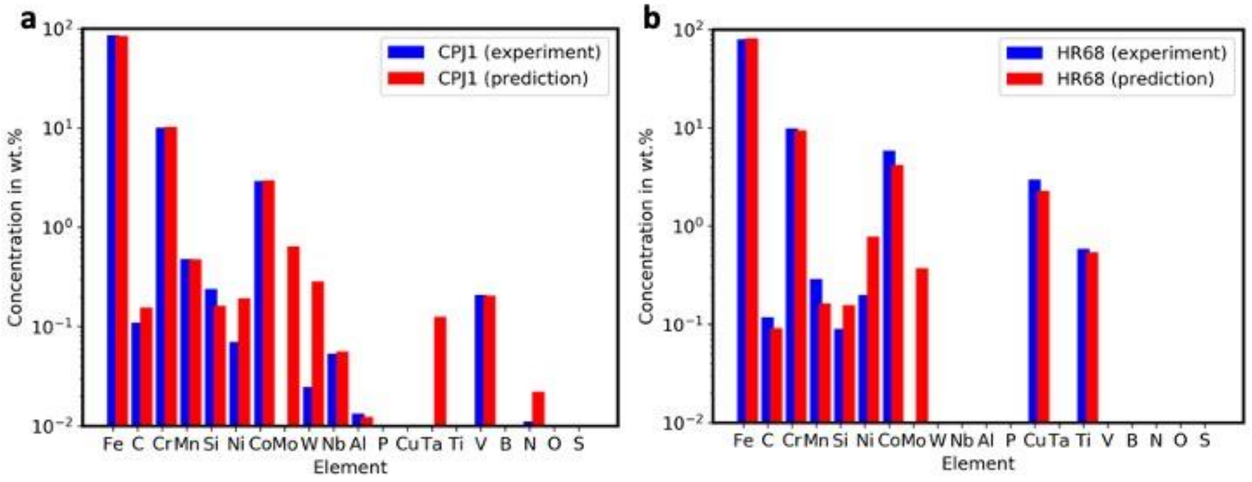

Figure S4. *Comparison between experimental and predicted concentrations for two alloys. A new model is trained without samples of CPJ1 and HR68, and then used to predict the concentrations of the two alloys. As is shown here, for both alloys, the model can well predict the experimental concentration, demonstrating the predictability of new alloys.*

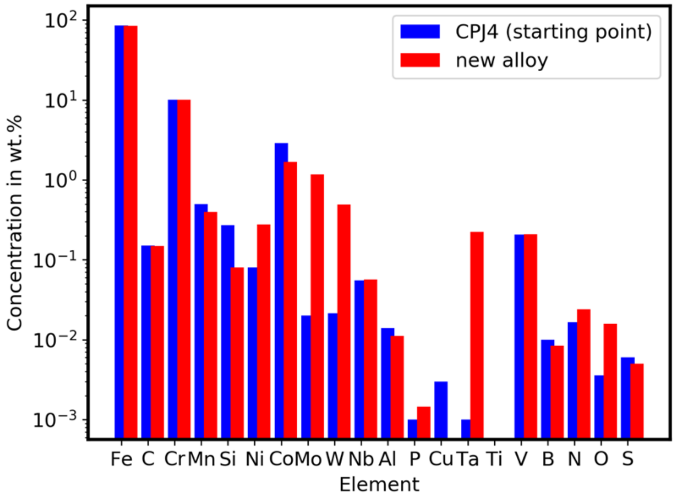

Figure S5. *A new alloy identified by the inverse design neural network. Starting from CPJ4, possible steels are explored with better mechanical performance with one exemplary candidate identified with noticeably higher contents of Mo, W and Ta.*

Supplementary information to the processing procedures

The steel ingots were made using vacuum induction furnace from high purity elemental charge materials (except for Ti, Fe-Ti alloy was added after the charge was molten). The processing routes were different for the CPJ and HR steels. CPJ ingots sequentially underwent homogenization, hot working at 1000°C, normalizing at 1150°C, and tempering at 700°C to develop a tempered martensitic microstructure [1]. HR ingots (HR 52-68) were thermo-mechanically processed at 740°C. Through a combination of forging, hot rolling, and reheating, the ingots were reduced to 12 mm thick plates in eight passes, achieving a ferritic microstructure.

References

[1] J.A. Hawk and P.D. Jablonski, Heat-Resistant Advanced 9% Steel for Fossil Energy Power Generation, TechConnect Briefs 2017, TechConnect.org, ISBN 978-0-9975117-9-6.
